# npInv: accurate detection and genotyping of inversions using long read sub-alignment

**DOI:** 10.1186/s12859-018-2252-9

**Published:** 2018-07-13

**Authors:** Haojing Shao, Devika Ganesamoorthy, Tania Duarte, Minh Duc Cao, Clive J. Hoggart, Lachlan J. M. Coin

**Affiliations:** 1 Genomics of Development and Disease Division, Institute for Molecular Bioscience, University of Queensland, 306 Carmody Rd, St Lucia, Brisbane, 4067 Australia; 2 Department of Medicine, Imperial College London, Level 2, Faculty Building South Kensington Campus, London, SW7 2AZ United Kingdom

**Keywords:** Inversion, Non allelic homologous recombination (NAHR), Inverted repeat (IR), Split read, Long read sequencing, NA12878

## Abstract

**Background:**

Detection of genomic inversions remains challenging. Many existing methods primarily target inzversions with a non repetitive breakpoint, leaving inverted repeat (IR) mediated non-allelic homologous recombination (NAHR) inversions largely unexplored.

**Result:**

We present npInv, a novel tool specifically for detecting and genotyping NAHR inversion using long read sub-alignment of long read sequencing data. We benchmark npInv with other tools in both simulation and real data. We use npInv to generate a whole-genome inversion map for NA12878 consisting of 30 NAHR inversions (of which 15 are novel), including all previously known NAHR mediated inversions in NA12878 with flanking IR less than 7kb. Our genotyping accuracy on this dataset was 94%. We used PCR to confirm the presence of two of these novel inversions. We show that there is a near linear relationship between the length of flanking IR and the minimum inversion size, without inverted repeats.

**Conclusion:**

The application of npInv shows high accuracy in both simulation and real data. The results give deeper insight into understanding inversion.

**Electronic supplementary material:**

The online version of this article (10.1186/s12859-018-2252-9) contains supplementary material, which is available to authorized users.

## Background

Inversion polymorphisms, in which the orientation of a segment of DNA is flipped with respect to its ancestral orientation relative to the rest of the chromosome, were originally discovered in 1917 by Sturtevant as a suppressor of recombination between chromosomes in hybrids of different strains of Drosophila [1]. Inversions can be broadly classified on the basis by which they are formed as non-homologous end joining (NHEJ [2]), non allelic homologous recombination (NAHR) or fork stalling and template switching (FoSTeS [3]) inversions. NHEJ is a pathway for repairing double-strand breaks in DNA. The inversion sequence ligates directly to breakpoint without large homologous sequence [2]. NAHR is an aberrant recombination mechanism which occurs between homologous sequences. Homologous recombination between inverted repeats (IRs) will invert the intervening sequence and create an inversion [4]. Almost all (12/14) known large inversion (> 1 Mb) polymorphisms are mediated by NAHR [5]. FoSTeS [3] is a DNA replication error resulting in multiple copies of local sequences in both forward and reverse order. Although FoSTeS generates inverted sequences, we prefer to classify FoSTeS inversion as a type of complex copy number variation rather than a simple inversion.

Inversion polymorphisms remain one of the most poorly mapped classes of genetic variation. Before the advent of sequencing, it was only possible to identify large cytogenetically visible inversions via microscopy [6]. Inversions can be detected from aberrant linkage disequilibrium (LD) patterns from population single-nucleotide polymorphism (SNP) genotyping data, but this provides limited power to detect inversions smaller than 500 kb or with minor allele frequency less than 25% [7–9]. Inversions can be inferred from second generation sequence data by abnormal pair end mapping and split read alignment [10]. In theory, this approach can be used to detect all NHEJ inversions [11]. Thus the remaining poorly understood inversions are NAHR inversions with inverted repeats longer than library insert size. These NAHR inversions are too long for short read and too short for the cytogenetic approach to be detected. Third generation sequencing platforms, in particular Oxford Nanopore Technologies can sequence reads up to hundreds of kilobases, which is suitable to span IR in order to detect NAHR inversion. To fill the gap of poorly known inversion, we design a new tool, namely npInv (nanopore Inversion), for use with third generation sequencing data to detect long NAHR inversions including those flanked by IRs, but not including complex events containing additional SVs.

## Results

### Detecting and genotyping inversion

**Fig. 1:**
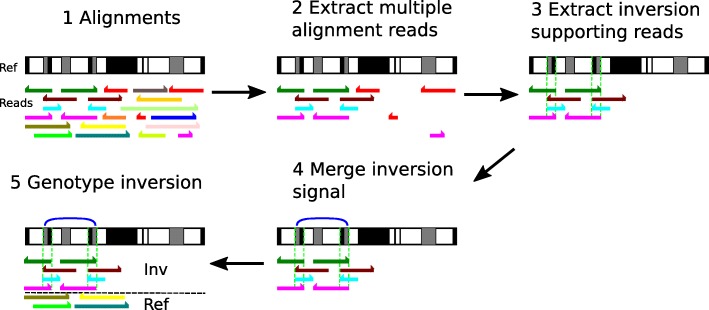
Software pipeline. The same colour bars indicate the alignment from the same reads. Half arrows indicate the orientation of the alignment. (1) The original alignment in a region. (2) Reads with multiple sub-alignments to the same chromosome are retained. Uniquely aligned reads are removed. (3) We obtain inversion signals and identify inverted repeats from sub-alignment. If inverted repeats (green dash lines) are observed, inversions are classified as NAHR, otherwise it is classified as NHEJ. Non-inversion information reads are removed. (4) Inversion signals were merged into regions as blue arcs. (5) Once the inversion regions are defined, we estimated the number of inversion reads as well as the number of reads supporting the non-inverted (reference) allele, which are removed in the step (2). Horizontal black dash line indicates the classification of inversion and reference reads. Finally, the software predicts the inversion with position, mechanism and genotype

**Fig. 2:**
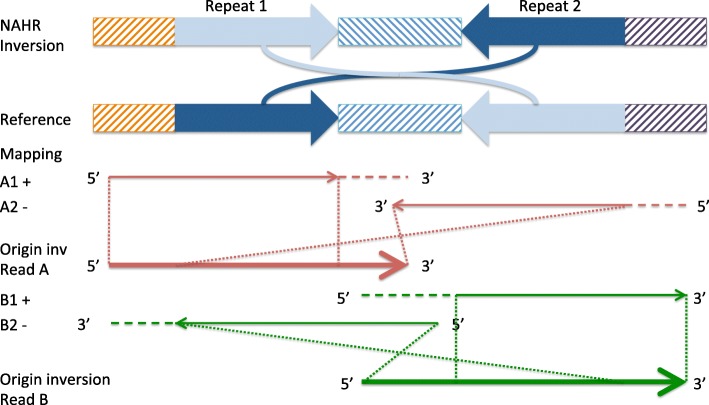
Illustration of the effect of a NAHR mediated inversion on long read sub-alignments. Idealised NAHR inversion and reference are shown in first two panels. Inverted repeats are shown as dark and light blue. Orange, dark purple and blue hashed rectangles indicate unique sequence. The direction of the hashing indicates its orientation. The third panel (red) shows a read supporting the left breakpoint of the inversion. The large arrow indicates the original unmapped read. The smaller arrows indicate two sub-read alignments, with the direction of the arrow indicating the alignment orientation, and the horizontal dashed line indicating aligned and clipped sequence. The dot lines indicate the position of the subread alignment on the original read. The fourth panel (green) is similar to the third panel, except that it illustrates the read supporting the right breakpoint

We present npInv, a novel tool designed specifically for detecting and genotyping NAHR mediated inversions from long read sequencing data. The input to npInv is an alignment file in bam format generated from local aligner such as BWA-MEM [12]. npInv’s pipeline and pseudo code are shown in Fig. 1 and in supplementary methods, respectively. In brief, npInv scans the alignment file for reads that contain pairs of subread alignments mapping to the same chromosome but with a different orientation (Fig. 2). npInv records this subread alignment pair as an inversion signal. If a subread alignment pair overlap in the original read, npInv records this overlapping sequence as an inverted repeat. npInv clusters and filters all the inversion signals in order to detect into inversion event based on position and the number of inversion signals in the cluster. npInv reports both the number of reads which support an inversion, as well as the number of reads supporting the non-inverted allele (reads which span the inversion breakpoints). Finally, npInv applies a binomial model [13] to genotype inversion from these read counts (see Methods). npInv reports the position, mechanism and genotype of each inversion.

### Benchmarking the software using simulated data

**Fig. 3:**
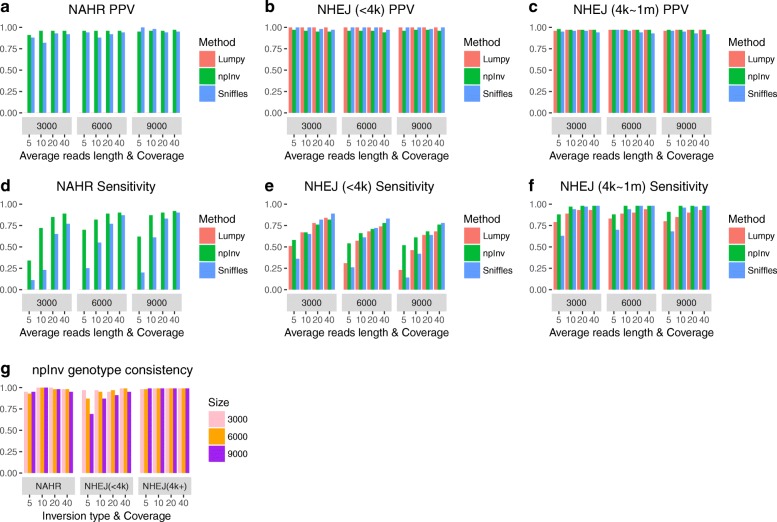
Performance comparison for npInv, Lumpy and Sniffles at three type of inversions. We simulated a diploid human chromosome 21 with three types of inversions: non-allelic homologous recombination (NAHR), non-homologous end joining (NHEJ) with size less than 4 kb and NHEJ with the size between 4 kb to 1 Mb (n = 61, 100 and 100, respectively). Software Lumpy [16], Sniffles [17] and npInv were applied to the above datasets. (**a**, **b**, **c**) and (**d**, **e**, **f**) showed Positive predicted value (PPV) and sensitivity for each method, respectively. (**g**) showed Genotype Consistency(GC) for npInv. Lumpy did not detect NAHR inversion and genotype information is not available for Lumpy or Sniffles

We first benchmarked the software using simulation data. We simulated 61 NAHR, 100 short (< 4 kb) and 100 long (> 4 kb) NHEJ non-overlapping inversions in reference GRCh37 chromosome 21. NAHR inversions were simulated based on the location of IR of length above 500 bp in the reference chromosome 21 (which limited their number to 61). We randomly set the genotype of inversion to be heterozygous or homozygous. Next, we used readsim [14] to simulate reads with an average read length of 3 kb, 6 kb or 9 kb. Sequence substitution, insertion and deletion rates were set at 5.1, 4.9 and 7.8*%*, respectively based on previously described characteristics of nanopore sequence data [15]. Sequence depth was set at 5, 10, 20 or 40 fold for different simulations. Reads were aligned by BWA-MEM [12]. The alignment result was used for npInv, as well as for software Lumpy [16] and Sniffles [17]. Lumpy was designed for detecting deletions, duplications, inversions and translocations specifically from second generation sequencing data. It used read-pair split-read and read depth signals to detect variations [16], however when applied to detecting inversions from single-end long-read data, only split-read signals will be informative. Sniffles is designed for detecting deletions, duplications, insertions, inversions, translocations as well as inversions flanked by deletion from long read sequencing data. It uses both main-alignment and sub-alignment to detect variations, as small indels can be aligned as gaps in main-alignment while larger or complex variations can only be aligned as multiple sub-alignments [17]. Both tools primarily focused on inversion without specific reference to the presence of IR. The positive predictive value (PPV, Fig. 3a-c), sensitivity (S, Fig. 3d-f) and genotyping consistency (GC, Fig. 3g) were calculated for each dataset.

For simulated NAHR inversions, npInv demonstrated substantially better sensitivity (41 to 210%)than the next best program (Sniffles) over all coverage and read-lengths simulated (Fig. 3a). Although Lumpy identified inversion breakpoints for simulated NAHR inversions as structural variation breakpoints, however it was unable to merge any of these breakpoints into a single inversion event. This likely reflects the limitations of a split-read alignment approach, which does not allow overlapping sub-read alignments. Allowing for overlapping sub-reads is important for reads which span an inverted repeat. Referring to Fig. 2, if a split-read approach was used, the A2 alignment on reverse strand would only comprise the unique right-most region within the inversion, and would not span the right breakpoint. The PPV of npInv was also highest across most coverage and read-lengths, although Sniffles’ PPV, which was slightly (2% to 5%) higher than npInv in low coverage long read datasets (Fig. 3a). npInv’s PPV remained high (> 90*%*) across all datasets, while its sensitivity depended on the depth and read length. npInvs’s sensitivity was good (> 80*%*) at 20 fold coverage and it did not improve significantly when the depth increased to 40 fold. On the other hands, Sniffles’ sensitivity improved constantly as the depth increased (Fig. 3d). This was because Sniffles required more supporting reads than npInv(default: Sniffles 10, npInv 3). The read length did not play a key role on both PPV and sensitivity, which was likely due to the fact that most of the background IR used to simulate NAHR inversions are of length less than the shortest average simulated read length (of 3 kb).

For NHEJ inversions the difference between the algorithms was not as pronounced. For long (> 4*kb*) NHEJ inversions, PPV for all 3 methods was more than 92% (Fig. 3c, 3f). The sensitivity of the three methods was similar (around 80%) for 20x coverage or higher, but npInv had a higher sensitivity at lower coverage (Fig. 3f). For short (< 4 kb) NHEJ inversions, the PPV for all 3 methods was higher than 94% (Fig. 3b), but their sensitivity ranged from 26 to 89% (Fig. 3e). Lumpy’s sensitivity was lower than previously reported using simulations of highly accurate short paired-end reads [16]. For all 3 tools, the sensitivity decreased with increasing average read length. This reflects limitations of existing alignment algorithms on long error-prone reads. When the aligners align long read data, they have to decrease the penalty for gap opening and extending in order to adapt the relatively high sequencing error rate in long read sequencing. As a result, aligners preferred to incorrectly align more sequence at the inversion breakpoint. Even worse, when the inversion was short compared to the read length, the aligner might fully align the inversion spanning read to the reference with wrong gap opening and extending at the inversion flipping sequence (Additional file 1: Figure S1). In this case, an inversion supporting read would be incorrectly regarded as a reference supporting read.

We further tested long read aligners (Minimap2 [18], NGMLR [17], BLASR [19] and GraphMap [20]) by npInv on simulated high depth(40X) long reads (9k) short NHEJ (< 4*kb*) inversion dataset, although npInv detected no inversion from BLASR and GraphMap alignments (see Methods). Minimap2 and NGMLR improved the PPV and sensitivity for inversion detection from (0.96,0.76) to (1,0.79) and (0.98,0.9) respectively although genotype consistency decreased from 0.95 to 0.59 and 0.88 (Additional file 1: Figure S2). We investigated this improvement by analyzing the mutation rates on a simulated 0.8 kb inversion (see Methods). We found mutation rates of Minimap2 and NGMLR were almost the same on both the inversion and its flanking regions, while the mutation rates of BWA-MEM, BLASR and GraphMap were higher at the inversion region (Additional file 1: Figure S3a, S3b, S3c). Minimap2 obtained higher insertion rate before or after inversion (Additional file 1: Figure S3c) and generated some long insertions (Additional file 1: Figure S3d). This suggested Minimap2 preferred to use one or more insertions and deletions to explain an inversion. NGMLR generated more alignment breakpoints at both inversion left and right breakpoints (Additional file 1: Figure S3e). This also suggested NGMLR preferred to chop an inversion around inversion breakpoints into multiple sub-alignments. Both approaches can generate more reliable inversion supporting reads, which improve the accuracy of npInv.

npInv was the only algorithm which reports the genotype for each inversion. To correctly genotype an inversion both the inversion read and reference read should be detected correctly. npInv’s genotype consistency was higher than 90% for long NHEJ inversions and NAHR inversions but was lower for short NHEJ inversions with low coverage and long reads (9kb) (Fig. 3g). The genotyping error is mainly caused by the limits of sensitivity in detecting reads supporting the inversion, and as a result counting these reads as reference-supporting, leading to homozygous inversions being annotated as heterozygous (Additional file 1: Figure S4). This was particularly a problem in conjunction with the issues regarding alignment to short inversions as discussed in the previous paragraph (Additional file 1: Figure S1).

### Benchmarking the software using real data

**Fig. 4:**
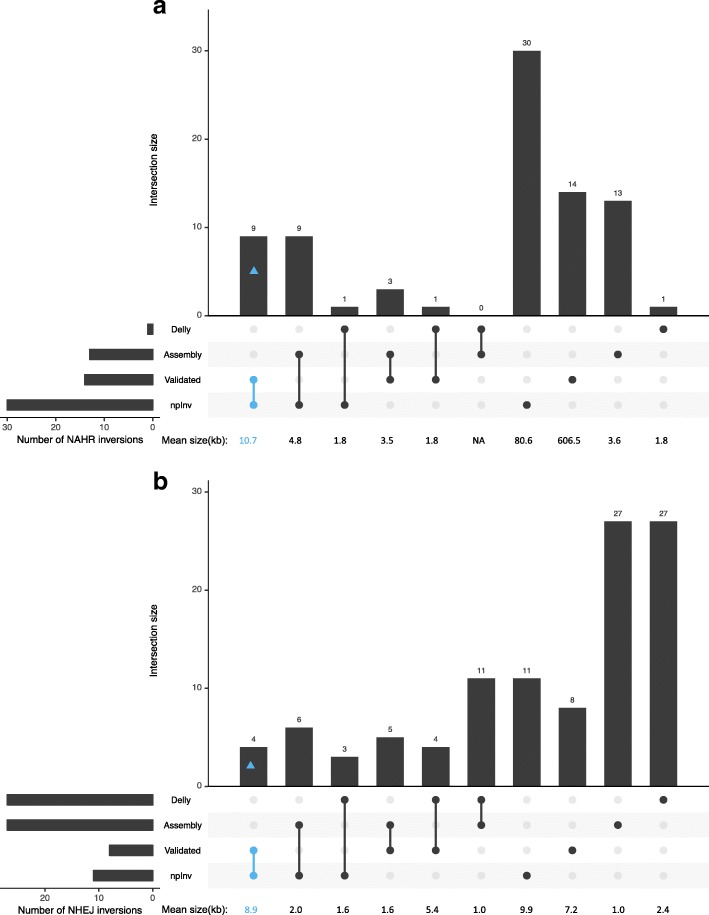
Intersection of four inversion datasets for individual NA12878. The four inversion datasets are labeled a. *Validated* (from InvFEST [5]); *Delly* (derived from Illumina sequence data by Sudmant, et al. [33]); *Assembly* (derived from a PacBio assembly by Pendleton, et al. [32] and *npInv*, derived from nanopore sequence data. (**a**) and (**b**) are for NAHR and NHEJ inversions, respectively. The number of inversions in the intersection is shown in the bar chart. The connected dots below the bar chart indicate which methods are included in each intersection. The mean size of inversions in the intersection is shown under each bar. Intersection containing both npInv and validated are highlight with blue. The total number of predicted inversion is shown on the bottom left. This figure was generated using ggplot2 [41] and modified version of UpSetR [42]

We aligned Nanopore high coverage human sequencing data on sample NA12878 [21] to GRCh37 and identified 41 inversions using npInv. We compared our results to a ’truth dataset’ of inversions from InvFest [5], which is a database of validated inversions using various techniques including fluorescent in situ hybridization (FISH), polymerase chain reaction (PCR) [22–31]. We also compared this result to short read sequencing result of NA12878 by Delly [10] and long read (Pacbio) assembly based on inversion call set [32] (Fig. 4). npInv detected 18 (15 NAHR, 3 NHEJ) novel inversions and 23 (15 NAHR, 8 NHEJ) inversions overlapping one or more dataset(InvFEST [5], Delly [10, 33] or Pacbio assembly [32]). As a truth dataset, InvFEST recorded 22(14 NAHR, 8 NHEJ) inversions, of which 13 (9 NAHR, 4 NHEJ) inversions were detected by npInv. npInv analysis of nanopore sequence data had the largest overlap with the validated dataset compared to the PacBio assembly (5) and Delly Illumina analysis (8). This is because npInv (mean inversion size 61 kb) can detect both short and long inversions, while assembly (mean 1.8 kb) and Delly (mean 2.3 kb) preferentially identify short inversions. Inversions from the InvFEST database which could not be detected by npInv include inversions shorter than 2 kb (3), flanked by IR longer than 7 kb (5) or inversion with a deletion (1). In other words, npInv could detect all nine detectable (IR < 7kb) validated NAHR inversion as well as four out of five validated NHEJ inversion with size > 2kb.

We also used a set of validated 36 inversion sites in NA18278 (derived from InvFEST) to validate genotype consistency of npInv. The genotype consistency for homozygous reference, heterozygous inversion and homozygous inversion are 100%(23/23), 83%(5/6) and 86%(6/7) in the real data, respectively. Overall it is 94%(34/36).

### Experimental validation of novel inversions

We selected three novel inversions of size > 1kb predicted by npInv which could be validated using a PCR based approach. As this requires a PCR product which spans the inverted repeat, this placed an upper limit on the size of the IR to be less than 2 kb. Three of 18 novel inversions passed these criteria predicted from npInv. We checked inversion 4q35.2 (NHEJ), 3q21.3 (NAHR) and 10q11.22 (NHEJ) by PCR (Additional file 1: Figure S5). Among these 3 inversions, there were 2 predicted heterozygous (4q35.2 and 3q21.3) and 1 homozygous inversion (10q11.22). We were able to validate predicted genotypes at two inversions. However, the 4q35.2 NHEJ inversion, could not be validated by PCR. Visual inspection of aligned nanopore reads revealed a clear structural variation breakpoint (Additional file 1: Figure S6 top) which was also predicted to be an inversion by Sniffles [17]. However, inspection of Pacbio [32] reads revealed almost no clipped reads, indicating an absence of an inversion (Additional file 1: Figure S6 bottom). Instead of sequencing error or mapping artefact, we surmise that the inversion observed in the nanopore sequence data may be due to a somatic mutation which occurred in a precursor cell to those used for Nanopore sequencing, however this is difficult to prove without access to the exact cell-line used in sequencing.

### Inversion map for NA12878

**Fig. 5:**
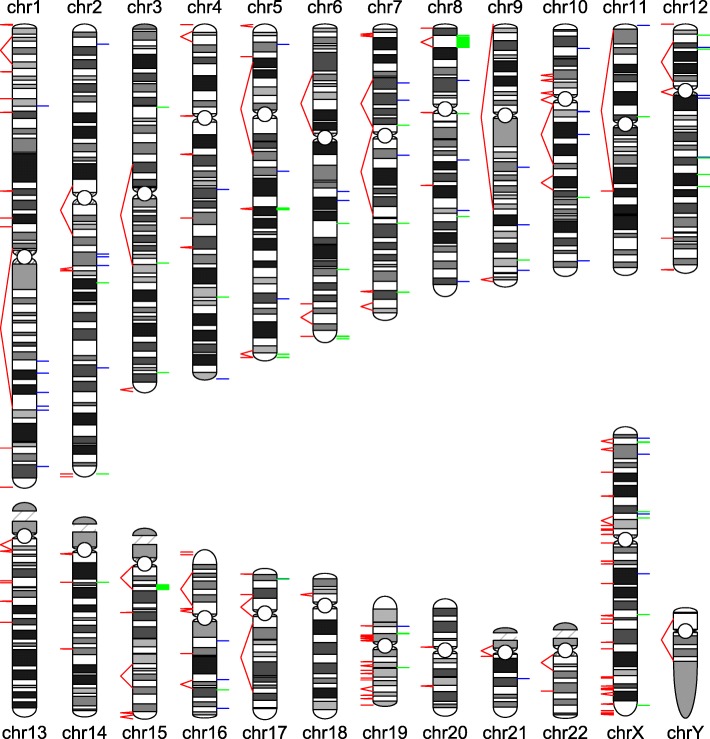
An inversion map for NA12878. A karyogram of human genome is depicted. The predicted (by existing methods) and potential (by genome compositions) inversions are shown on the right and left of the chromosome, respectively. Green bars are NAHR or palindrome inversions. Blue bars are NHEJ or FoSTeS inversions. Red line pairs indicate inverted repeat pairs which may mediate NAHR inversions. The bars and line pairs will be seen as a line if the distance between them is short

**Fig. 6:**
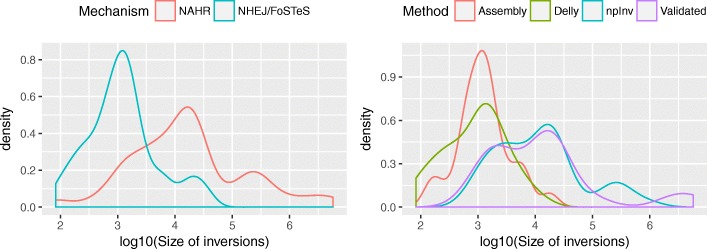
Inversion length distribution in density **a** Length distribution for NAHR and NHEJ/FoSTeS inversion. We broadly regard the non NAHR inversion as NHEJ/FoSTeS inversion. **b** Length distribution for Delly [10, 33], Assembly [32], npInv and Validated [5, 22–31] datasets. Density was estimated by function density in R

We combined all inversions detected by four different approaches on NA12878 including Delly applied to Illumina sequence data [33], Pendleton et al. applied to Pacbio and Bionano sequence data [32], InvFest database of validated inversions [5], as well as novel inversions discovered by npInv. This resulted in a set of 87 known inversions, which we mapped to a karyogram (Fig. 5). We observed that NAHR inversions (mean size 275kbp) are longer than NHEJ or FoSTeS inversions (3.8 kb) (Fig. 6). Short read methods like Delly [10] primarily focus on NHEJ inversion or NAHR inversion for which IR size is shorter than library insert size. Thus, it mainly reports the distribution of NHEJ inversions. On the other hand, the long read splitting method at IR like npInv could extend the range of detection to longer NAHR inversion (Fig. 6).

We classified inversions according to the size of flanking IR as short (< 500bp), median (500-7000bp) and long (> 7kb) (Table 1). Short IR inversions can be detected by PEM based methods from short read sequencing data and local assembly [34], particularly as the local sequence structure is typically not repetitive around short variants. Median IR inversions are efficiently detected using npInv as shown above.

**Table 1:** Classification of inversions and their solution: assuming the short read sequencing insert size is 500 bp

Class	Inverted repeat size(bp)	Inversion size(bp)	Type of inversion	Efficient detection method	Number of inversions
Short	[0,500]’	[2,INF)	NHEJ/FoSTeS, NAHR	Pair end mapping/Local assembly	52
Median	(500,7000]	(1000,INF)	NAHR	Split long read	23
Long	(7000,INF)	(14000,INF)	NAHR	Microscope, other	12

INF means infinity

### Characteristics of NAHR inversions

**Fig. 7:**
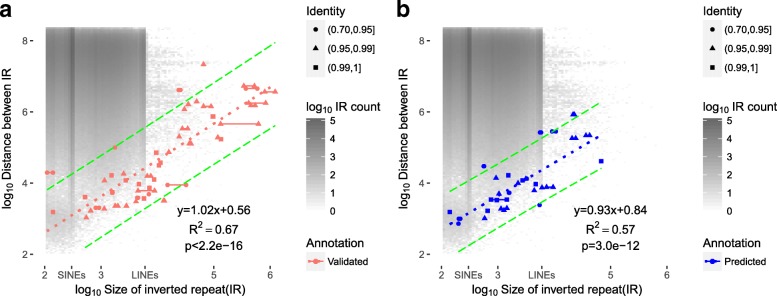
Relationship between inverted repeat (IR) length and distance between repeats. (**a**) is from 50 validated NAHR inversions from invFest and (**b**) is from 30 predicted NAHR inversions at NA12878 using npInv. Two points are drawn for each pair of IR which mediates a NAHR inversion. In the majority of cases the pair of IRs have similar length and so the two points are co-localised; otherwise the two points are connected by a line. The distance between repeats in NAHR inversion is the inversions size without repeats. The y-axis indicates the distance between the IRs and the x-axis indicates the length of the IR. The dotted line indicates the linear regression from log10 average size of IR to log10 size of inversion without flanking IR. The green long dash lines are 90% prediction intervals. The slope, intercept, R-squared and t-test *p*-value for linear regression are shown at bottom right. The background heat map indicates the count of IR in the genome with given IR length and distance between IR. The size of short interspersed nuclear elements (SINEs, 300 bp) and long interspersed nuclear elements(LINEs, 7 kb) is shown in the x-axis legend

We investigated the relationship between IR and NAHR inversion by summarizing all the background IR in the genome as well as predicted and validated NAHR inversions (Fig. 7, see Methods). The background IRs mainly occur with length less than 10 kb and between repeat distance ranging from 10 Mb to 100 Mb. There are two hotspots for IR at around 300bp and 6000bp, which is mainly due to the random distribution of short interspersed nuclear elements (SINEs) and long interspersed nuclear elements (LINEs) in the chromosome. If the probability of a NAHR inversion occurring is equal amongst all the IRs, the distribution of NAHR inversion should be the same as the distribution of background IR. However, we found the NAHR inversion distribution is totally different from the background IR distribution. Surprisingly, there is an almost linear relationship between the size of inverted repeat and the inversion (*p*-value < 1e-10), as well as an apparent empirical upper and lower bound on the size of an IR (90% prediction interval, green long dashed lines in Fig. 7) which can mediate an inversion of a certain size. For example, a 1Mb inversion can only be mediated by an IR of length greater than 50kb. This suggests only some IRs have the potential to mediate non-allelic homologous recombination and become a NAHR inversion. For larger (IR > 50 kb) NAHR inversions, the size of first and second inverted repeat are not always the same and the identity could be lower (0.90 to 0.99). As the size of IR increases, the tolerance of recombination also increases.

We use this observed relationship to map the potential location of inversion mediated by large IR (> 7kb) in the human genome, which is still not well characterized by sequencing based approaches. We filter all IR pairs with length greater than 7kb on the basis of the distance between the repeats (see Methods) to identify IR pairs which can mediate inversions (Fig. 7). This leaves 140 regions in which large IR inversions could occur (Fig. 5 and Additional file 1: Table). All of the 5 known NAHR inversions with IR greater than 7kb observed in NA18278 occur within one of these regions.

## Discussion and conclusion

We developed a new tool, npInv, to detect and genotype inversion from long read sequencing data, with particular application to data generated using Oxford Nanopore sequencing technologies devices. The application of npInv shows high accuracy in both simulation and real data. We use npInv to uncover an almost linear relationship between inverted repeat and NAHR inversion and show the potential of providing an individual inversion map. With the possible widespread adoption of long read sequencing data, application of npInv could help extend our understanding of the extent of inversion polymorphism, their evolutionary significance and their clinical impact.

We report the most comprehensive whole-genome inversion map to date, consisting of 87 inversions, of which 38 are NAHR mediated inversions and the remained are NHEJ or FOSTES mediated. The ability of this approach to detect an NAHR mediated inversions is limited by the depth of coverage of sequence reads of length greater than the size of the flanking inverted repeat. The data we used has 6.8X coverage of fragments of 7kb or longer and we were able to detect all validated inversions in NA12878 of length less than 7kb. This indicates that 7x coverage of reads greater than *X*kb is the minimum required to detect inversions flanked by repeats of *X*kb. There are a further 2130 IRs with length above 7 kb, which may not be detectable using this dataset. We reduced the number of IR by exploiting the knowledge of potential sites of NAHR inversion and merging the overlapping IRs. We finally identified a further potential 140 inversion loci with IR length greater than 7 kb (see Methods). An increase in the yield of ultra-long (> 100 kb) sequence data on this sample, coupled with algorithmic improvements in alignment of long reads will help refine the location of inversions flanked by these long IR.

Detecting inversions from long reads requires a different approach comparing to short reads. The key process of long read inversion detection is finding the correct sub-read alignment using long and high-error sequencing data while the key process of short read inversion detection is correct combination of read-pair information and split-read alignment using high accuracy sequencing data. We have demonstrated that using a split-read approach commonly used by short-read SV detection algorithms is less sensitive, particularly with respect to detecting inversions flanked by inverted repeats than using sub-reads. Both Sniffles and npInv identify inversions primarily by looking for subread alignments with different orientation. Sniffles identifies inversions as part of a process of identifying multiple classes of SV, whereas npInv is purely focused on the detection of inversions. Both Sniffles and npInv have similar performance on high coverage long-read datasets; however by optimising parameters for inversion detection, npInv is able to achieve higher sensitivity on lower coverage long-read sequencing datasets without sacrificing specificity. npInv also uses information on the overlap of sub-read alignments to annotate inversions as NHEJ or NAHR mediated inversions.

## Methods

### Inverted repeat mapping

Different size of inverted repeats required different methods. Long (> 1*kb*) inverted repeat were identified by extracting inverted duplications from SD database [4] at http://humanparalogy.gs.washington.edu/. This contains long inverted repeats with long (> 10*kb*) insertions or deletions. Median(> 500*bp*) inverted repeat were identified by running inverted repeat finder [35](IRF, version 3.05) for each chromosome. The parameter was 2 3 5 80 10 800 50000 300000 -d -h -t4 1000 -t5 10000 -t7 300000. Short (> 100*bp*) inverted repeats were identified by using last [36](v458) to align each chromosome to its self. Each alignment pair with identity greater than 0.90 was defined as an inverted repeat pair. The parameter was -s 0 (reversed alignment).

For Fig. 5, we first filtered the IR (inferred from SD database [4]) less than 7k to identify 2130 IRs. We carried out a linear regression of IR length against the inversion size on all InvFEST and all npInv detected inversions in NA12878 respectively. The regression parameters obtained are similar. We use the InvFEST regression parameters to build a predictive model of the length of IR against the distance between IR (i.e. the minimum potential inversion size). We removed the IRs outside of 90% prediction interval by R to identify 1302 IRs. Then we applied BEDTools [37] to sort and merge these regions into 140 non-overlapping genomic regions.

### Analysis of long read sub-alignment

npInv focuses on reads which have multiple sub-alignments. For each of these sub-read alignments, i, we sort the alignments by its left-most location in the read. Then we record the ($start^{i}_{read}$, $end^{i}_{read}$) co-ordinates of the alignment on the read, the ($start^{i}_{ref}$, $end^{i}_{ref}$) co-ordinates in the reference genome, as well as the reference orientation and chromosome. For a read containing multiple sub-alignments (Fig. 2), we perform the following analysis. We first filter alignments with length less than 500 bps or for which the alignment interval on the read is totally contained by another alignment interval. Next, for each pair of read-adjacent alignment intervals (which are allowed to overlap), we keep pairs mapping to the same chromosome and in different alignment orientation as potential inversion signals (A1-A2, B1-B2 in Fig. 2). If the first sub-alignment is in forward strand, we record this signal as an inversion forward supporting signal. Otherwise, we record as reverse signal. If the first alignment’s location in reference is bigger than the second alignment, we record this signal as a left breakpoint inversion supporting signal. Otherwise, we record this signal as right. If two alignment intervals are overlapping by more than 500bp (on the read), this inversion signal is considered to be mediated by NAHR. The overlapping sequence could align to the inverted repeat in both orientations (light blue and dark blue in Fig. 2). Only one of these pair of read alignments includes sequence from the inversion itself (Fig. 2). All inversion signals are sorted by chromosome and left-most start position on the reference.

### Analysis of inversion signal

After scanning the bam file by split read analysis, the software identifies numerous inversion signals. The user has the option of providing a database of known IR pairs in the genome. If this is provided, the software creates a bin for each IR in the database which is used to merge inversion signals. Each bin represents an inversion call. For each candidate inversion, we check whether the left breakpoint and right breakpoint are within X bp (in practice, X=2000) in the IR database’s left and right repeat sequence. If true, group them in this IR bin and delete the binned signal. If the user does not provide an IR database, and also for the remaining signals which cannot be clustered around known IR, inversion signals are grouped into the same bin if their reference start and end are both less than X bp (default 2000) from each other. We then investigate whether each merged inversion signal contains supporting reads on both forward and reverse strands, and also at both left and right breakpoints. The output inversion’s start and end are the mean value of the left breakpoints and right breakpoints, respectively. The output inversion left and right breakpoint start and end are the minimum and maximum alignment position at left and right breakpoint in the reference, respectively. We calculate the inversion supporting read *R*_*inv*_ as the sum of reads supporting the left and right breakpoint signal for genotyping inversion. If a read supports both left and right breakpoint, it will count as one left breakpoint signal and one right breakpoint signal.

npInv annotates the inversion longer than *L*(parameter, default 1Mb) as long inversion. We consider that long inversion is not reliable for either NHEJ (usually shorter than 1 Mb) or NAHR (likely with inverted repeat which is too long to be fully spanned) inversion.

### Analysis of non-inversion signal

We calculate the average substitution, deletion and insertion rate and their standard deviations from the first min(10000,*all*) primary alignments. For each alignment overlapping with inversion, we define its inversion region as (max(left breakpoint start, alignment start), min(right breakpoint end, alignment end)). We calculate three error rates (substitution, deletion and insertion rate) in its inversion region from the primary alignments. If the all three error rates are less than its average rate plus its one standard deviation, we kept this alignment as a reference supporting alignment. We calculate the sum of reference supporting read *R*_*ref*_ for the next step. If a read spans both left and right breakpoint and passes the criteria, it will count as one reference left breakpoint signal and one reference right breakpoint signal.

### Inversion genotyping

For each binned inversion, we get the number of inversion and reference supporting reads (*R*_*inv*_,*R*_*ref*_) from the above step. Applying binomial model [13] on the genotyping inversion, the posterior probability *P* of genotype *G*=*g*_*refref*_,*g*_*refinv*_,*g*_*invInv*_ conditional on the observed read counts *R*_*ref*_ and *R*_*inv*_ could be written as below.

1 $$ P(G|R_{ref},R_{inv}) \propto P(R_{ref},R_{inv}|G)P(G)  $$

The likelihood *P*(*R*_*ref*_,*R*_*inv*_|*G*) could be written as

2 $$ {{P(R_{Ref},R_{Inv}|G)\! \propto\!\! \left\{\!\begin{array}{ll} (1-\epsilon_{1})^{R_{ref}}\epsilon_{2}^{R_{inv}}, & G=g_{refref} \\ \left(\frac{1-\epsilon_{1}}{2}+\frac{\epsilon_{2}}{2}\right)^{R_{ref}}\left(\frac{\epsilon_{1}}{2}+\frac{1-\epsilon_{2}}{2}\right)^{R_{inv}}, & G=g_{refinv} \\ \epsilon_{1}^{R_{ref}}(1-\epsilon_{2})^{R_{inv}}, & G=g_{refinv} \\ \end{array}\right. \!,}}  $$

where *ε*_1_ and *ε*_2_ are the error rates of incorrectly assigning an inversion-supporting read to a reference supporting read and vice-versa, respectively(in practice, we use *ε*_1_=*ε*_2_=0.01, however with availability of more data it would be possible to infer specific mis-assignment rates). We assume an uniform prior such that *P*(*G*=*g*_*inv*_)=*P*(*G*=*g*_*ref*_)=0.5 and then by Hardy-Weinberg equilibrium *P*(*G*=*g*_*refref*_)=*P*(*G*=*g*_*invInv*_)=0.25 and *P*(*G*=*g*_*refinv*_)=0.5. Then we choose the maximum posterior probability genotype as the genotype for the individual. The genotype quality Q is calculated as the second maximum posterior probability *P*_2*nd*_ divided by the maximum posterior probability *P*_1*st*_ in Phred quality score as below.

3 $$ Q=-10{log}_{10}\frac{P_{2nd}}{P_{1st}}  $$

### Inversion simulation and benchmarking

We chose the whole GRCh37 chromosome 21 as the reference. We grouped the inversions into three types, which were NAHR, short (0-4 kb) NHEJ and long (4 kb to 1 Mb) NHEJ inversions. We simulated 61 NAHR, 100 short and 100 long NHEJ non-overlapping inversions in reference chromosome 21. NAHR inversions were simulated based on the reference IR (> 500 bp) from IRF [35] and limited to 61 non-overlapping NAHR inversions on chromosome 21. We randomly set the genotype of inversion as heterozygous or homozygous. Then we simulated a diploid chromosome 21 and flipped over the simulated inversion interval in one or two chromosomes according to its genotype. Next, we used readsim [14] (version 1.6) to simulate reads from this diploid chromosome with an average read length of 3 kb, 6 kb or 9 kb. Sequence substitution, insertion and deletion rates were set at 5.1, 4.9 and 7.8*%*, respectively based on previously described characteristics of nanopore sequence data [15]. Sequence depth was set at 5, 10, 20 or 40 folds for different simulations. The readsim parameter is sim fa –rev_strd on –tech nanopore –read_mu 3000,6000,9000 –read_dist exp –cov_mu 5,10,20,40 –err_sub_mu 0.051 –err_in_mu 0.049 –err_del_mu 0.078.

Simulation reads were aligned by BWA-MEM [12] (version 0.7.15-r1142-dirty) to chromosome 21. The BWA-MEM parameter is -t 16 -x ont2d -M, which is suggested by Sniffles’ readme. The alignment result was used for npInv (version 1.2), as well as for software Lumpy [16] and Sniffles [17]. We run Lumpy (v0.2.13) from its executable file named lumpy with parameter -mw 4 -tt 1e-3 -sr bam_file:BAMINPUT,back_distance:20,weight:1,id:1,min_mapping_threshold:1. Sniffles (version 1.0.5) was downloaded from https://github.com/fritzsedlazeck/Sniffles. We applied Sniffles directly to the simulation bam files with the default parameter. Lumpy or Sniffles inversions were called when their vcf ALT fields are equal to <*INV*>. An inversion was classified as positive predictive inversion when the true simulation inversion interval was 90% overlapping with the predictive inversion interval, and vice-versa. Finally, the positive predictive value (PPV), sensitivity (S) and genotype consistency (GC) were calculated for different datasets.

We aligned high depth(40X) long read(9000 bp) short NHEJ(< 4*kb*) inversion simulation dataset by Minimap2 [18](version 2.9-r720), NGMLR [17](version 0.2.7), GraphMap [20](version v0.5.2) and BLASR [19](version 1.3.1). The Minimap2 parameter is “-t 15 -k15 -w5 –splice -g2000 -G200k -A1 -B2 -O2,32 -E1,0 -C9 -z200 -ub –splice-flank =yes”. The NGMLR parameter is “-x ont”. The GraphMap and BLASR parameters are both default.

Mutation rates are calculated in each 100 bps windows. Insertion rate are estimated as the total size of insertion sequences divided by the total size of aligned sequences (no insertion and deletion) in each 100 bps windows. (Insertion rate may more than 1 for regions containing large insertions.) Substitutions, insertions and deletions are extracted by samtools [38] mpileup(version:1.3.1, parameter:default).

### Classification of inversion by mechanism

For inversions from InvFEST, we accepted the mechanism from InvFEST. For the remaining unclassified inversions, we checked whether the start and end were within inverted repeats from the SD database [4]. If an inverted repeat was found, we classified the inversion as NAHR mediated with IR sizes and identity from SD database. Otherwise, we extracted the whole inversion sequence and aligned it to itself by YASS [39]. If the YASS’s dotplot showed inverted repeat sequence at both the start and the end, we classified it into NAHR inversion. The IR sizes and identity were determined by the YASS’s alignment result. When the inversion was totally reverse complement, we classified it as Palindrome. We classified the remaining inversions into NHEJ/FoSTeS inversion.

### Detection of inversion on NA12878

NA12878 raw data [21](version rel3) was downloaded from https://github.com/nanopore-wgs-consortium/NA12878. We aligned it to GRCh37 by BWA-MEM [12] (version 0.7.15-r1142-dirty). The key parameter was -k11 -W20 -r10 -A1 -B1 -O1 -E1 -L0 -Y. We ran npInv(version 1.2) with default parameter. The predicted inversions are the inversions whose “FILTER” field is equal to “PASS” in vcf [40] file.

### PCR validation

PCR was used to validate 3 inversions detected from the sequencing data. Two forward primers were designed to overlap the inversion breakpoints, one to amplify the reference copy and a second primer to amplify the inverted copy with a shared reverse primer. PCR reactions were performed using 1x HotStar Taq DNA Polymerase (Qiagen), 2.5mM MgCl_2_, 200nM of forward primer (either to amplify the reference or the inverted sequence), 200nM reverse primer and 2ng/uL of DNA NA12878. PCR conditions were optimized for each PCR target. The following PCR conditions were used: hot start at 95 °C for 15 minutes, 35 cycles of 95 °C for 30s, 60 °C for 30s and 72 °C for 4 minutes with a final extension of 10 minutes at 72 °C. An annealing temperature of 55 °C was used to amplify the inverted sequence of 3q21.3. PCR products were analyzed by horizontal electrophoresis on 1.5% agarose gel.

## Additional file

Additional file 1 **Supplementary Figure. Figure S1.** Error rates distribution around a wrong mapping homozygous inversion region. **Figure S2.** Evaluation of BWA-MEM [1], Minimap2 [2] and NGMLR [3] using npInv with short NHEJ inversions. **Figure S3**. Error rates distribution of 5 aligners. **Figure S4.** The performance of genotyping inversion from simulated and real data. **Figure S5.** PCR products validating three inversions (4q35.2, 3q21.3 and 10q11.22). **Figure S6.** IGV [7] view for left breakpoint on inversion 4q35.2. **Supplementary Information. Algorithm 1.** Program pseudocode. **Supplementary Table. Table S1.** PCR primers for validation. **Table S2.** NA12878 inversion combined from npInv, Validated [6] (Val), Assembly [8] (Asse) and Delly [9]. (PDF 325 kb)

## Abbreviations

FISH: Fluorescent in situ hybridization
FoSTeS: Fork stalling and template switching
GC: Genotyping consistency
IR: Inverted repeat
IRF: Inverted repeat finder
LD: Linkage disequilibrium
LINEs: Long interspersed nuclear elements
NAHR: Non-allelic homologous recombination
NHEJ: Non-homologous end joining
npInv: Nanopore inversion
PCR: Polymerase chain reaction
PPV: Positive predictive value
SINEs: Short interspersed nuclear elements
SNP: Single-nucleotide polymorphism

## References

[1] Sturtevant AH (1917). Genetic factors affecting the strength of linkage in drosophila. Proc Natl Acad Sci.

[2] McVey M, Lee SE (2008). Mmej repair of double-strand breaks (director’s cut): deleted sequences and alternative endings. Trends Genet.

[3] Zhang F, Khajavi M, Connolly AM, Towne CF, Batish SD, Lupski JR (2009). The dna replication fostes/mmbir mechanism can generate genomic, genic and exonic complex rearrangements in humans. Nat Genet.

[4] Bailey JA, Eichler EE (2006). Primate segmental duplications: crucibles of evolution, diversity and disease. Nat Rev Genet.

[5] Martínez-Fundichely A, Casillas S, Egea R, Ràmia M, Barbadilla A, Pantano L, Puig M, Caceres M (2013). Invfest, a database integrating information of polymorphic inversions in the human genome. Nucleic Acids Res.

[6] Feuk L, Carson AR, Scherer SW (2006). Structural variation in the human genome. Nat Rev Genet.

[7] Bansal V, Bashir A, Bafna V (2007). Evidence for large inversion polymorphisms in the human genome from hapmap data. Genome Res.

[8] Cáceres A, Sindi SS, Raphael BJ, Cáceres M, González JR (2012). Identification of polymorphic inversions from genotypes. BMC Bioinformatics.

[9] Sindi SS, Raphael BJ (2010). Identification and frequency estimation of inversion polymorphisms from haplotype data. J Comput Biol.

[10] Rausch T, Zichner T, Schlattl A, Stütz AM, Benes V, Korbel JO (2012). Delly: structural variant discovery by integrated paired-end and split-read analysis. Bioinformatics.

[11] Lledó JIL, Cáceres M (2013). On the power and the systematic biases of the detection of chromosomal inversions by paired-end genome sequencing. PLoS One.

[12] Li H. Aligning sequence reads, clone sequences and assembly contigs with bwa-mem. 2013. Preprint. https://arxiv.org/pdf/1303.3997.pdf.

[13] Shao H, Bellos E, Yin H, Liu X, Zou J, Li Y, Wang J, Coin LJ (2012). A population model for genotyping indels from next-generation sequence data. Nucleic Acids Res.

[14] Richter DC, Ott F, Auch AF, Schmid R, Huson DH (2008). MetaSim–A sequencing simulator for genomics and metagenomics. PLoS ONE.

[15] Jain M, Fiddes IT, Miga KH, Olsen HE, Paten B, Akeson M (2015). Improved data analysis for the minion nanopore sequencer. Nat Methods.

[16] Layer RM, Chiang C, Quinlan AR, Hall IM (2014). Lumpy: a probabilistic framework for structural variant discovery. Genome Biol.

[17] Sedlazeck FJ, Rescheneder P, Smolka M, Fang H, Nattestad M, von Haeseler A, Schatz M (2018). Accurate detection of complex structural variations using single molecule sequencing. Nature Methods.

[18] Li H. Minimap2: fast pairwise alignment for long dna sequences. 2017. Preprint. https://arxiv.org/pdf/1708.01492.pdf.

[19] Chaisson MJ, Tesler G (2012). Mapping single molecule sequencing reads using basic local alignment with successive refinement (blasr): application and theory. BMC Bioinformatics.

[20] Sović I, Šikić M, Wilm A, Fenlon SN, Chen S, Nagarajan N (2016). Fast and sensitive mapping of nanopore sequencing reads with graphmap. Nat Commun.

[21] Jain M, Koren S, Quick J, Rand AC, Sasani TA, Tyson JR, Beggs AD, Dilthey AT, Fiddes IT, Malla S (2018). Nanopore sequencing and assembly of a human genome with ultra-long reads. Nat Biotechnol.

[22] Korbel JO, Urban AE, Affourtit JP, Godwin B, Grubert F, Simons JF, Kim PM, Palejev D, Carriero NJ, Du L (2007). Paired-end mapping reveals extensive structural variation in the human genome. Science.

[23] Kidd JM, Cooper GM, Donahue WF, Hayden HS, Sampas N, Graves T, Hansen N, Teague B, Alkan C, Antonacci F (2008). Mapping and sequencing of structural variation from eight human genomes. Nature.

[24] Pang AW, MacDonald JR, Pinto D, Wei J, Rafiq MA, Conrad DF, Park H, Hurles ME, Lee C, Venter JC (2010). Towards a comprehensive structural variation map of an individual human genome. Genome Biol.

[25] Wang J, Wang W, Li R, Li Y, Tian G, Goodman L, Fan W, Zhang J, Li J, Zhang J (2008). The diploid genome sequence of an asian individual. Nature.

[26] Ahn S-M, Kim T-H, Lee S, Kim D, Ghang H, Kim D-S, Kim B-C, Kim S-Y, Kim W-Y, Kim C (2009). The first korean genome sequence and analysis: full genome sequencing for a socio-ethnic group. Genome Res.

[27] McKernan KJ, Peckham HE, Costa GL, McLaughlin SF, Fu Y, Tsung EF, Clouser CR, Duncan C, Ichikawa JK, Lee CC (2009). Sequence and structural variation in a human genome uncovered by short-read, massively parallel ligation sequencing using two-base encoding. Genome Res.

[28] Stefansson H, Helgason A, Thorleifsson G, Steinthorsdottir V, Masson G, Barnard J, Baker A, Jonasdottir A, Ingason A, Gudnadottir VG (2005). A common inversion under selection in europeans. Nat Genet.

[29] Giglio S, Calvari V, Gregato G, Gimelli G, Camanini S, Giorda R, Ragusa A, Guerneri S, Selicorni A, Stumm M (2002). Heterozygous submicroscopic inversions involving olfactory receptor–gene clusters mediate the recurrent t (4; 8)(p16; p23) translocation. Am J Hum Genet.

[30] Osborne LR, Li M, Pober B, Chitayat D, Bodurtha J, Mandel A, Costa T, Grebe T, Cox S, Tsui L-C (2001). A 1.5 million–base pair inversion polymorphism in families with williams-beuren syndrome. Nat Genet.

[31] Gimelli G, Pujana MA, Patricelli MG, Russo S, Giardino D, Larizza L, Cheung J, Armengol L, Schinzel A, Estivill X (2003). Genomic inversions of human chromosome 15q11–q13 in mothers of angelman syndrome patients with class ii (bp2/3) deletions. Hum Mol Genet.

[32] Pendleton M, Sebra R, Pang AWC, Ummat A, Franzen O, Rausch T, Stütz AM, Stedman W, Anantharaman T, Hastie A (2015). Assembly and diploid architecture of an individual human genome via single-molecule technologies. Nat Methods.

[33] Sudmant PH, Rausch T, Gardner EJ, Handsaker RE, Abyzov A, Huddleston J, Zhang Y, Ye K, Jun G, Fritz MH-Y (2015). An integrated map of structural variation in 2,504 human genomes. Nature.

[34] Treangen TJ, Salzberg SL (2012). Repetitive dna and next-generation sequencing: computational challenges and solutions. Nat Rev Genet.

[35] Warburton PE, Giordano J, Cheung F, Gelfand Y, Benson G (2004). Inverted repeat structure of the human genome: the x-chromosome contains a preponderance of large, highly homologous inverted repeats that contain testes genes. Genome Res.

[36] Kiełbasa SM, Wan R, Sato K, Horton P, Frith MC (2011). Adaptive seeds tame genomic sequence comparison. Genome Res.

[37] Quinlan AR (2014). Bedtools: the swiss-army tool for genome feature analysis. Curr Protoc Bioinforma.

[38] Li H, Handsaker B, Wysoker A, Fennell T, Ruan J, Homer N, Marth G, Abecasis G, Durbin R (2009). The sequence alignment/map format and samtools. Bioinformatics.

[39] Noé L, Kucherov G (2005). Yass: enhancing the sensitivity of dna similarity search. Nucleic Acids Res.

[40] Danecek P, Auton A, Abecasis G, Albers CA, Banks E, DePristo MA, Handsaker RE, Lunter G, Marth GT, Sherry ST, et a.l (2011). The variant call format and vcftools. Bioinformatics.

[41] Wickham H. Ggplot2: Elegant Graphics for Data Analysis. Springer; 2016. https://www.springer.com/gp/book/9783319242750.

[42] Conway JR, Lex A, Gehlenborg N (2017). Upsetr: An r package for the visualization of intersecting sets and their properties. Bioinformatics.

