# Supplementary material for: npInv: accurate detection and genotyping of inversions using long read sub-alignment
Source: BMC Bioinformatics. 2018 Jul 13;19:261. doi: 10.1186/s12859-018-2252-9 (PMC6044046; doi:10.1186/s12859-018-2252-9)
Supplement: Supplementary file 1 — Supplementary Figure. Figure S1. Error rates distribution around a wrong mapping homozygous inversion region. Figure S2. Evaluation of BWA-MEM [1], Minimap2 [2] and NGMLR [3] using npInv with short NHEJ inversions. Figure S3. Error rates distribution of 5 aligners. Figure S4. The performance of genotyping inversion from simulated and real data. Figure S5. PCR products validating three inversions (4q35.2, 3q21.3 and 10q11.22). Figure S6. IGV [7] view for left breakpoint on inversion 4q35.2. Supplementary Information. Algorithm 1. Program pseudocode. Supplementary Table. Table S1. PCR primers for validation. Table S2. NA12878 inversion combined from npInv, Validated [6] (Val), Assembly [8] (Asse) and Delly [9]. (PDF 325 kb) [file 12859_2018_2252_MOESM1_ESM.pdf]

1 Supplementary Figure

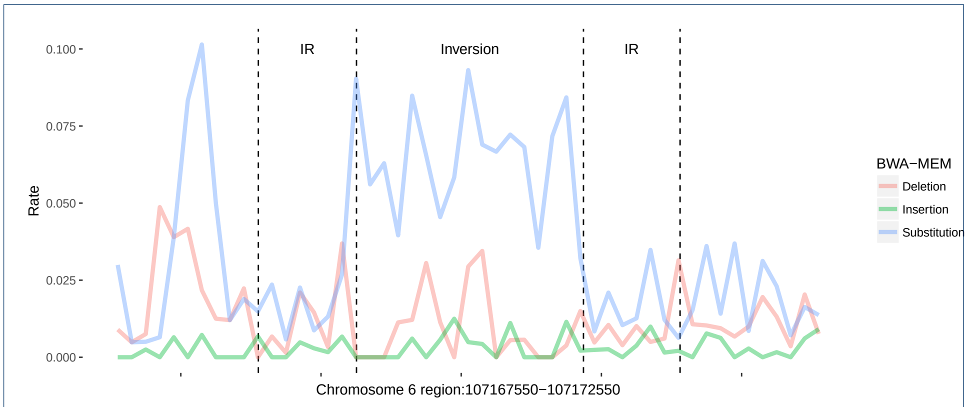

Figure S1: **Error rates distribution around a wrong mapping homozygous inversion region.** The Nanopore data is aligned by BWA-MEM to a known inversion(1.6 kb) with inverted repeats(IR, 0.7 kb). The border of inverted repeat (IR) and inversion are showed as vertical lines. Substitution, deletion and insertion rate are estimated from all the alignments in this region. Many alignments are spanning the whole inversion regions (figure not show). The high error rate within inversion indicates wrong alignments.

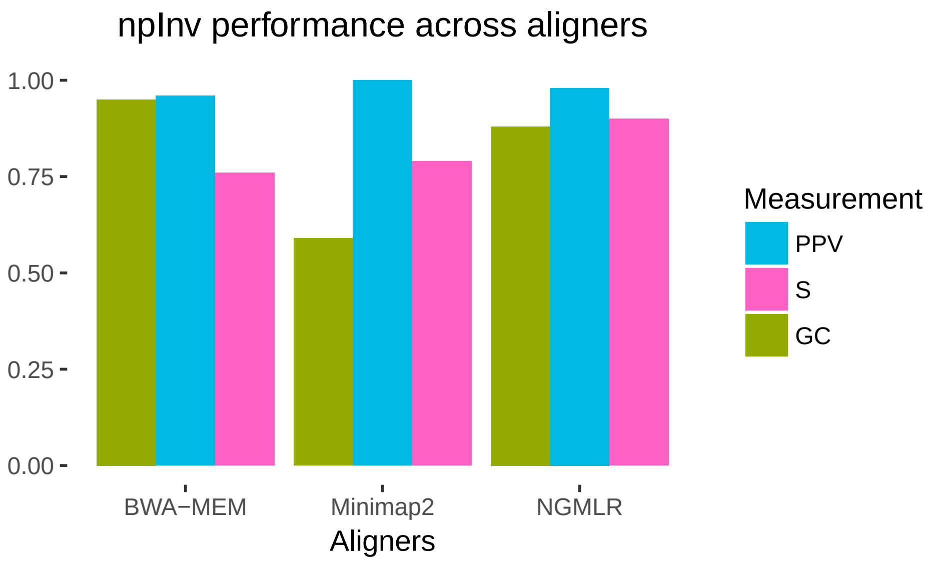

Figure S2: **Evaluation of BWA-MEM[1], Minimap2[2] and NGMLR[3] using npInv with short NHEJ inversions** npInv is applied to alignment from BWA-MEM, Minimap2 and NGMLR on high depth(40X) long read(9000 bp) short NHEJ($< 4kb$) inversion simulation dataset. PPV: Positive predictive value. S: Sensitivity. GC: Genotype consistency.

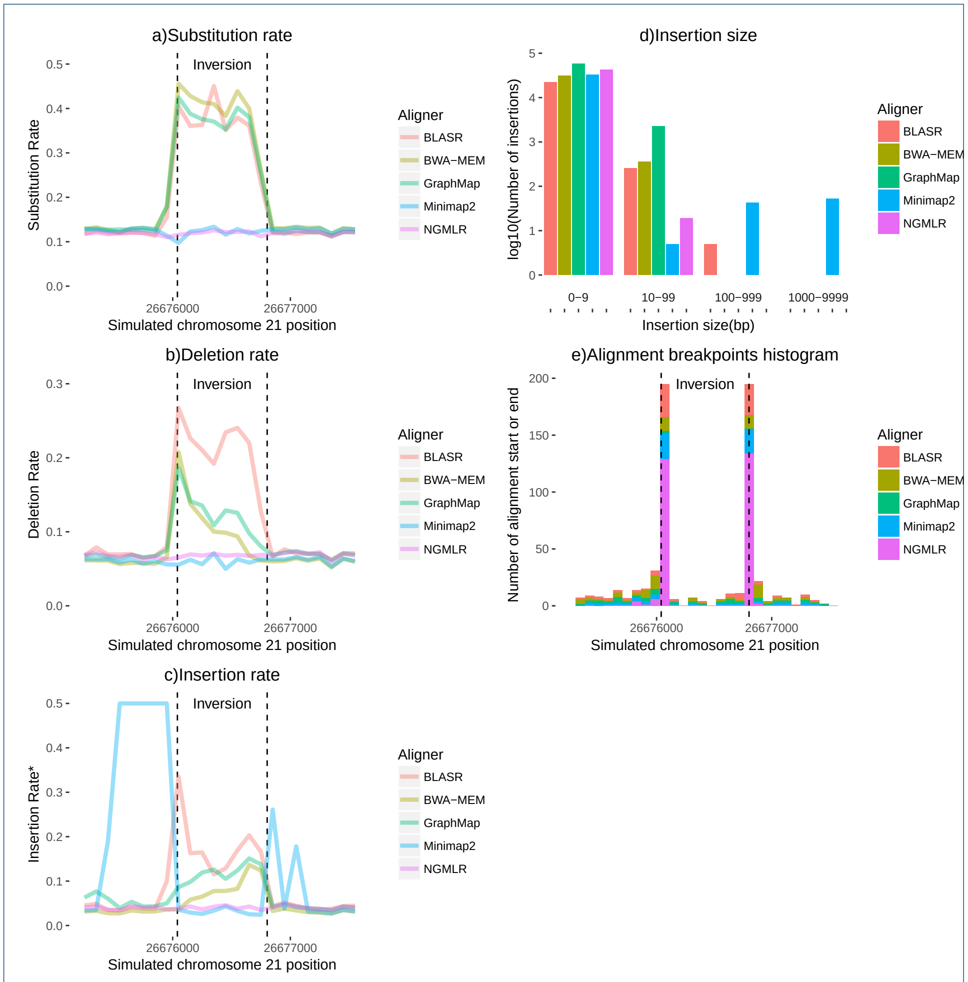

Figure S3: **Error rates distribution of 5 aligners.** BLASR[4], BWA-MEM[1], GraphMap[5], Minimap2[2] and NGMLR[3] align simulated long reads (9 kb) to a simulated short inversion(0.8 kb). The inversion breakpoints are shown as vertical dashed lines. **a**, **b** and **c** are substitution, deletion and insertion rate distribution for each 100 bps window around the inversion(see Methods). *****: Insertion rate more than 0.5 are downscaled to 0.5. **d**, Insertion size distribution in a logarithmic scale. Minimap2 generates long size insertions while other aligners do not. **e**, Alignment breakpoints histogram. NGMLR generates more breakpoints around inversion breakpoints comparing to other aligners.

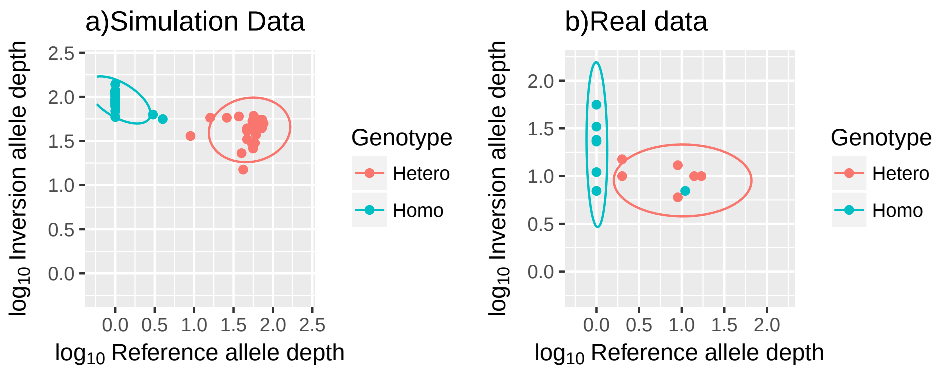

Figure S4: **The performance of genotyping inversion from simulated and real data.** Each dot is an inversion. X and Y axises are the base 10 logarithm of the estimated reference and inversion allele depth, respectively. "Hetero" and "Homo" are for heterozygous and homozygous inversion, respectively. Reference and inversion allele depth were estimated by npInv from (a) simulation data (40 folds and average read length 6 kb NAHR inversion) and (b) real data (NA12878 nanopore data unpublished). The genotype represented in the legend is the true genotype in the simulation (a) or validated database (b)[6], respectively. The coloured ellipse indicates high confidence genotype prediction by npInv.

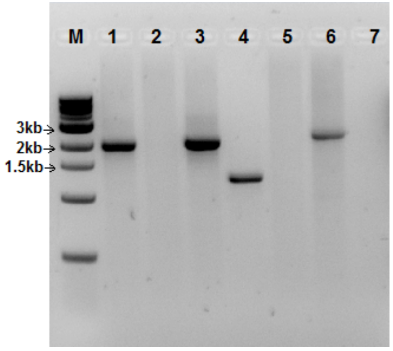

Figure S5: **PCR products validating three inversions(4q35.2, 3q21.3 and 10q11.22).** A band indicates this sequence exists in the genome. Lane1, 4q35.2 non-inverted. Lane2, 4q35.2 inverted. 4q35.2 is predicted to be heterozygous. Lane3, 3q21.3 non-inverted. Lane4, 3q21.3 inverted. 3q21.3 is predicted to be heterozygous. Lane5, 10q11.22 non-inverted. Lane6, 10q11.22 inverted. 10q11.22 is predicted to be homozygous. Lane7, Non template control. Primers could be found at supplementary material.

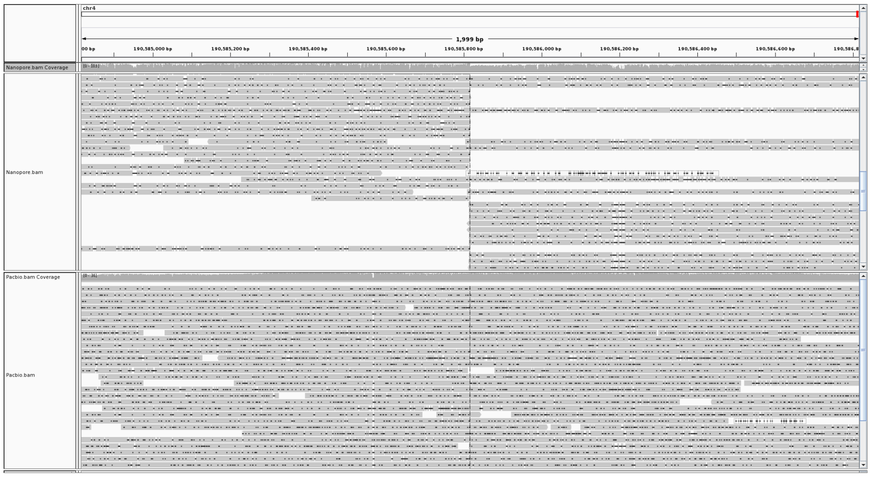

Figure S6: **IGV[7] view for left breakpoint on inversion 4q35.2.** The top and bottom bam file are from Nanopore (unpublished) and Pacbio[8], respectively. Each horizontal line is an aligned read. The middle vertical line on top indicates the left breakpoint for the NHEJ inversion.

2 Supplementary Information

Algorithm 1 Program pseudocode.

Input: alignment in bam format
Output: inversions *INV* in vcf format
for all reads in bam **do**
  if alignments > 1 **then**
    for all alignment $\subset$ this read **do**
      if alignment $\not\subset$ other alignment *and* alignment $>$ L *and* alignment chromosome $=$ adjacent alignment chromosome *and* alignment orientation $\neq$ adjacent alignment orientation **then**
        inv.firstBreakpoint=alignment position
        inv.secondBreakpoint=adjacent alignment position
        if alignment $\cap$ adjacent alignment $\neq \Phi$ **then**
          inv.invertedRepeat=alignment $\cap$ adjacent alignment
        end if
        $InvSignal_i$=inv
        i=i+1
      end if
    end for
  end if
end for
for all $inv \in$ *InvSignal* **do**
  if $inv_i \subseteq$ *IR* **then**
    $IR_j.signal_k = inv_i$
    k=k+1
    delete inv_i from *InvSignal*
  end if
end for
for all $inv \in$ *InvSignal* **do**
  if $inv_i \subseteq$ *INV* **then**
    $INV_j.signal_k = inv_i$
    k=k+1
  else
    j=j+1
    create INV_j
    $INV_j.singal_0 = inv_i$
  end if
end for
INV $=$ *INV* $\cup$ *IR*
for all $inv \in INV$ **do**
  if inv_i has signal from both first and second breakpoint *and* inv_i has signal from both forward and reverse strand **then**
    GENOTYPE(inv_i)
  else
    delete inv_i
  end if
end for
output *INV*

L=500, in practice. Initially, i, j and k=0.

3 Supplementary Table

Table S1: **PCR primers for validation.** Primer ID contains chromosome ID, location and ID. The product of primer A and primer B are for non inverted sequence. The product of primer A and primer C are for inverted sequence.

Primer ID	primer seq
4q35.2A	AGAATGTATTTTTAGTTCTCTGGAA
4q35.2B	GCACTTTAACCTACTGAGGAAT
4q35.2C	CGTGCCATGCTCTATGTACc
3q21.3A	CTTGAACAGATGCCTCACGA
3q21.3B	GTTGCTTTTCTTTGCATTTATGTT
3q21.3C	GGGCATGTTCCTTCCCTAC
10q11.22A	GGGATCCTCTGATGGACAAG
10q11.22B	CATGTGTCCTGGCTCTAGTG
10q11.22C	CCACTGCAACTCCATGTCAG

Table S2: **NA12878 inversion combined from npInv, Validated[6](Val), Assembly[8](Asse) and Delly[9].** "N","Y","0-1" and "1-1" mean no inversion, inversion with no genotype, heterozygous inversion and homozygous inversion, respectively. Inversion size is including the inverted repeat(IR) size. "@" means this inversion contains deletion. The reference version is GRCh37.

Chr	Start	End	npInv	Val	Asse	Delly	Mechanism	IR	Inv Size
chr1	44059289	44059889	N	N	Y	1—1	NHEJ/FoSTeS	0	601
chr1	181043910	181044074	N	N	Y	N	NHEJ/FoSTeS	0	165
chr1	187464823	187466729	N	N	Y	N	NHEJ/FoSTeS	0	1907
chr1	197756782	197757999	0—1	N	Y	0—1	NHEJ/FoSTeS	0	1218
chr1	205178631	205178779	N	N	Y	0—1	NHEJ/FoSTeS	0	149
chr1	207292347	207292429	N	N	Y	N	NHEJ/FoSTeS	0	83
chr1	237566104	237566206	N	N	Y	N	NHEJ/FoSTeS	0	103
chr2	10825964	10827217	N	N	N	0—1	NHEJ/FoSTeS	0	1254
chr2	123476599	123482503	N	N	N	0—1	NHEJ/FoSTeS	0	5905
chr2	125051701	125053265	N	N	N	0—1	NHEJ/FoSTeS	0	1565
chr2	129685049	129686125	N	N	N	0—1	NHEJ/FoSTeS	0	1077
chr2	139004622	139008966	1—1	N	Y	N	NAHR	1098	5637
chr2	184569169	184572006	0—1	0—1	Y	N	NHEJ/FoSTeS	0	2838
chr2	241615561	241638174	N	0—1	N	N	NAHR	13059	29296
chr3	44741074	44742201	1—1	N	Y	N	NAHR	200	1128
chr3	128346174	128376684	0—1	N	N	N	NAHR	565	31075
chr3	187133742	187144394	0—1	N	N	N	NAHR	4420	15072
chr4	88847163	88858700	0—1	0—1	N	N	NHEJ/FoSTeS	0	11538
chr4	146614721	146615901	N	N	Y	N	NAHR	360	1181
chr4	190585823	190613128	0—1	N	N	N	NHEJ/FoSTeS	0	27306
chr5	10906438	10908184	N	N	N	0—1	NHEJ/FoSTeS	0	1747
chr5	79046344	79049460	N	N	N	0—1	NHEJ/FoSTeS	0	3117
chr5	98858883	99727999	0—1	N	N	N	NAHR	22139	887305
chr5	147553039	147554615	N	1—1	Y	1—1	NHEJ/FoSTeS	0	1577
chr5	177209108	177434702	0—1	N	N	N	NAHR	39518	297150
chr5	179061247	179085461	0—1	N	N	N	NAHR	9924	24215
chr6	89923631	89923942	N	N	Y	N	NHEJ/FoSTeS	0	312
chr6	94741066	94741416	N	N	Y	N	NHEJ/FoSTeS	0	351
chr6	107168548	107171533	1—1	1—1	Y	N	NAHR	658	2984
chr6	131884820	131884921	N	N	Y	N	Palindrome	42	84
chr6	167581349	167804046	0—1	N	N	N	NAHR	22404	226869
chr6	169093045	169095237	1—1	1—1	Y	N	Palindrome	1097	2194
chr7	31586765	31592019	N	N	N	0—1	NHEJ/FoSTeS	0	5255
chr7	40879358	40880171	0—1	0—1	Y	0—1	NHEJ/FoSTeS	0	814
chr7	54290974	54386821	N	1—1	N	N	NAHR	15882	106144
chr7	70420968@	70438887	N	1—1	N	0—1	NHEJ/FoSTeS	0	17920
chr7	107058553	107063655	1—1	1—1	Y	N	NAHR	961	5271
chr7	143898767	144046496	0—1	N	N	N	NHEJ/FoSTeS	53000	147730

chr8	6154239	6158433	1—1	N	Y	N	Palindrome	2098	4196
chr8	6922488	12573597	N	1—1	N	N	NAHR	1012583	5708071
chr8	30245127	30246097	0—1	N	N	N	NHEJ/FoSTeS	0	971
chr8	48212887	48232801	0—1	N	N	N	NAHR	3320	19915
chr8	73023621	73023864	N	N	Y	N	NHEJ/FoSTeS	0	244
chr8	100157613	100158316	N	N	N	0—1	NHEJ/FoSTeS	0	658
chr8	103435524	103435965	N	N	Y	N	Palindrome	218	436
chr8	138348193	138349910	N	N	N	0—1	NHEJ/FoSTeS	0	1718
chr9	76898138	76898390	N	N	Y	N	NHEJ/FoSTeS	0	253
chr9	107816812	107817348	N	N	N	1—1	NHEJ/FoSTeS	0	537
chr9	126738652	126756051	1—1	1—1	N	N	NAHR	2734	17400
chr9	132186289	132186727	N	N	Y	N	NHEJ/FoSTeS	0	439
chr10	13104977	13105272	N	N	Y	0—1	NHEJ/FoSTeS	0	296
chr10	47022964	47059768	1—1	N	N	N	NHEJ/FoSTeS	0	36805
chr10	59256944	59257961	N	N	Y	N	NHEJ/FoSTeS	0	1018
chr10	93200597	93208532	0—1	N	Y	N	NAHR	5499	13434
chr10	127190584	127197225	N	N	N	1—1	NHEJ/FoSTeS	0	6642
chr11	738418	740631	0—1	N	Y	N	NHEJ/FoSTeS	860	2214
chr11	49734984	49751316	0—1	N	N	N	NAHR	3136	19468
chr12	6038269	6041280	0—1	N	N	N	NAHR	1284	4295
chr12	12544370	12547226	0—1	N	Y	1—1	NHEJ/FoSTeS	625	2857
chr12	13545034	13551091	1—1	N	Y	N	NAHR	1047	7105
chr12	38317979	38318554	N	N	N	1—1	NHEJ/FoSTeS	0	576
chr12	39860127	39860299	N	N	Y	0—1	NHEJ/FoSTeS	0	173
chr12	71532784	71533816	N	N	N	0—1	NHEJ/FoSTeS	0	1033
chr12	71939834	71941663	1—1	0—1	N	0—1	NAHR	140	1830
chr12	80844919	80859036	0—1	1—1	N	N	NAHR	6067	19640
chr12	87240472	87252688	1—1	N	N	N	NAHR	2281	14497
chr14	35010051	35031476	N	0—1	N	N	NAHR	7196	21782
chr15	30370112	32899708	N	1—1	N	N	NAHR	497247	2529597
chr16	48905267	48906227	N	N	Y	1—1	NHEJ/FoSTeS	0	961
chr16	69761804	69762886	N	N	N	0—1	NHEJ/FoSTeS	0	1083
chr16	75239338	75257419	0—1	0—1	N	N	NAHR	1534	19628
chr16	85188714	85189804	N	1—1	Y	0—1	NHEJ/FoSTeS	211	1500
chr17	5594699	5595567	N	N	N	0—1	NHEJ/FoSTeS	0	869
chr17	5885622	5886899	N	N	Y	N	Palindrome	608	1216
chr19	16167946	16168601	N	N	Y	N	NHEJ/FoSTeS	0	656
chr19	19890521	20160204	0—1	N	N	N	NAHR	5714	277546
chr19	38343057	38344968	1—1	N	Y	N	NAHR	280	1420
chr21	27374110	27374706	N	N	Y	1—1	NHEJ/FoSTeS	0	597
chrX	6137036	6138404	N	0—1	Y	N	NHEJ/FoSTeS	0	1369
chrX	8137458	8434752	0—1	N	N	N	NAHR	9914	303929
chrX	14729539	14731318	0—1	N	Y	N	NHEJ/FoSTeS	0	1780

chrX	45547772	45551190	1—1	N	N	N	NAHR	1446	4864
chrX	46810694	46830781	1—1	1—1	N	N	NHEJ/FoSTeS	0	20088
chrX	49013019	49019888	1—1	1—1	N	N	NAHR	1586	8529
chrX	78921883	78923381	N	N	Y	N	NHEJ/FoSTeS	0	1499
chrX	100852522	100871235	0—1	0—1	N	N	NAHR	4690	18782
chrX	149570890	149585747	1—1	N	N	N	NAHR	929	15786

Table S3: **Large potential inversion regions**

Chr	Start	End	Chr	Start	End	Chr	Start	End
chr1	92387	669292	chr10	29571648	30995196	chr19	41314896	41643680
chr1	6487720	21813075	chr10	36216349	37923219	chr19	43840402	43901918
chr1	25594515	25751819	chr10	38291975	43141400	chr19	44890506	44977572
chr1	39975198	40242350	chr10	43189721	75497421	chr19	48406743	51126682
chr1	47304216	47622817	chr10	81260743	89260363	chr19	52989727	53285299
chr1	89509514	89902910	chr11	3480151	89654671	chr19	54773980	55157672
chr1	104113370	104317467	chr11	89657025	89800177	chr19	58328232	58431914
chr1	108764242	109015286	chr12	60000	154905	chr20	25733092	26084413
chr1	120531870	206171611	chr12	9436203	31333164	chr20	46453805	47137605
chr1	227673690	227700791	chr12	34315477	38599805	chr21	9473009	15336945
chr1	248584239	248834181	chr12	114970358	115013262	chr21	15346934	15441979
chr2	86935956	113207451	chr12	131752588	132169194	chr22	17005667	25736039
chr2	130735169	132394768	chr13	19762373	25555912	chr22	36620662	36665122
chr2	132519150	132558354	chr13	25572706	26652632	chrX	6445602	8987338
chr2	241611923	241641219	chr13	42002895	42026163	chrX	11232454	13063191
chr2	243088899	243189373	chr13	43021708	43090111	chrX	24326510	24385773
chr3	75468544	129931618	chr13	52747106	53214381	chrX	36986405	37454830
chr3	195349462	197394388	chr13	64290924	64418388	chrX	47968524	52850418
chr4	299491	484589	chr14	19361502	20194548	chrX	52903909	53006000
chr4	3883064	9758387	chr14	35009872	35031654	chrX	54800000	54869088
chr4	49154264	49637650	chr14	70696492	70938383	chrX	55479962	55546105
chr4	69621553	70317435	chr15	20318547	32899708	chrX	57600296	57956094
chr4	104155256	104316686	chr15	45109634	45375390	chrX	62347592	62470907
chr4	119512833	120375873	chr15	72902298	85815511	chrX	71955011	72210791
chr5	235806	1636329	chr15	98643650	99553851	chrX	72215903	72306797
chr5	17517109	17599695	chr15	100319313	102334597	chrX	73535889	73719959
chr5	20695327	70660316	chr16	1278547	1308599	chrX	84099075	84139890
chr5	98726330	99736930	chr16	2584142	2727514	chrX	101051749	101720939
chr5	175329459	178949098	chr16	12018586	30328024	chrX	103172644	103358289
chr5	179060980	179085567	chr16	31570307	31809013	chrX	105489776	105545939
chr6	26667216	58726206	chr16	31961153	33864470	chrX	115575919	115814436

chr6	150231667	150348049	chr16	55758243	55873429	chrX	119172167	119332013
chr6	153754955	161319197	chr16	69977498	74590161	chrX	134250050	134390361
chr6	167579270	167806139	chr17	2954154	3156719	chrX	134729094	134823566
chr7	5007876	5888982	chr17	13923279	14144696	chrX	134846411	135032064
chr7	5933268	6872518	chr17	15422953	26090284	chrX	139086368	139311560
chr7	29692579	55795516	chr17	29363977	66268794	chrX	140077191	140789063
chr7	56244369	102445087	chr18	10604201	12231380	chrX	140997268	141281185
chr7	143218861	144074376	chr19	15762546	15997984	chrX	142596664	144337620
chr7	149586782	153864361	chr19	20741649	20966010	chrX	148613958	149116021
chr8	2180177	2343982	chr19	21426751	21559345	chrX	152331842	152349894
chr8	6933359	12542602	chr19	21820510	21928126	chrX	152371586	152560433
chr8	47459626	47663748	chr19	22441880	22993371	chrX	153564285	153624563
chr8	86552568	86841882	chr19	23487634	24173318	chrX	153783899	153877056
chr9	104099	99999015	chr19	35028657	35237078	chrX	154109089	154734211
chr9	136079723	138442756	chr19	36758541	37827104	chrY	6102852	28457769
chr10	26871864	28289317	chr19	39732381	39763662			

Author details

References

1. Li, H.: Aligning sequence reads, clone sequences and assembly contigs with bwa-mem. arXiv preprint arXiv:1303.3997 (2013)
2. Li, H.: Minimap2: fast pairwise alignment for long dna sequences. arXiv preprint arXiv:1708.01492 (2017)
3. Sedlazeck, F.J., Rescheneder, P., Smolka, M., Fang, H., Nattestad, M., von Haeseler, A., Schatz, M.: Accurate detection of complex structural variations using single molecule sequencing. bioRxiv, 169557 (2017)
4. Chaisson, M.J., Tesler, G.: Mapping single molecule sequencing reads using basic local alignment with successive refinement (blasr): application and theory. BMC bioinformatics **13**(1), 238 (2012)
5. Sović, I., Šikić, M., Wilm, A., Fenlon, S.N., Chen, S., Nagarajan, N.: Fast and sensitive mapping of nanopore sequencing reads with graphmap. Nature communications **7**, 11307 (2016)
6. Martínez-Fundichely, A., Casillas, S., Egea, R., Ràmia, M., Barbadilla, A., Pantano, L., Puig, M., Caceres, M.: Invfest, a database integrating information of polymorphic inversions in the human genome. Nucleic acids research, 1122 (2013)
7. Robinson, J.T., Thorvaldsdóttir, H., Winckler, W., Guttman, M., Lander, E.S., Getz, G., Mesirov, J.P.: Integrative genomics viewer. Nature biotechnology **29**(1), 24–26 (2011)
8. Pendleton, M., Sebra, R., Pang, A.W.C., Ummat, A., Franzen, O., Rausch, T., Stütz, A.M., Stedman, W., Anantharaman, T., Hastie, A., *et al.*: Assembly and diploid architecture of an individual human genome via single-molecule technologies. Nature methods **12**(8), 780–786 (2015)
9. Rausch, T., Zichner, T., Schlattl, A., Stütz, A.M., Benes, V., Korbel, J.O.: Delly: structural variant discovery by integrated paired-end and split-read analysis. Bioinformatics **28**(18), 333–339 (2012)
